# Unresectable Retiform Hemangioendothelioma Treated with External Beam Radiation Therapy and Chemotherapy: A Case Report and Review of the Literature

**DOI:** 10.1155/2010/756246

**Published:** 2010-09-26

**Authors:** Alina Z. Hirsh, Weisi Yan, Lihong Wei, A. Gabriella Wernicke, Bhupesh Parashar

**Affiliations:** ^1^Stich Radiation Center, Weill Cornell Medical Center, New York, NY 10065, USA; ^2^Department of Hematology/Oncology, New York Hospital Queens, NY 11355, USA

## Abstract

Retiform hemangioendothelioma (RH) is an infrequently encountered vascular neoplasm of intermediate or borderline malignancy. Treatment of RH is controversial. We present a case of a 44-year-old Asian male presenting with an unresectable RH of the pelvis. The patient was treated with concurrent low-dose Cisplatin and External beam Radiation (4140cGy in 180cGy per fraction). This is the first report of a clinical complete response and a long-term local control of this rare tumor. This has significant clinical implication, since it gives the first evidence of treatment of this rare tumor using concurrent low-dose chemotherapy and radiation.

## 1. Introduction

Retiform hemangioendothelioma (RH) is an infrequently encountered vascular neoplasm of intermediate or borderline malignancy. Treatment of RH is controversial though most reports have described surgical resection followed by adjuvant radiation (RT) with or without chemotherapy. We describe a case report of a 44-year-old Asian male presenting with an unresectable RH of the pelvis and successful treated with concurrent chemotherapy and radiation.

## 2. Case Report

**Figure 1:**
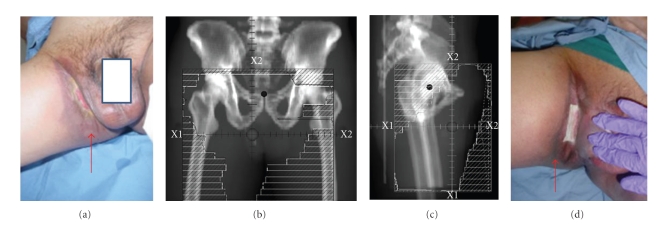
A 44-year-old male presenting with advanced RH of the pelvis and groin (a) Prior to treatment, arrow showing the extensive area of discoloration and skin breakdown (b) Anteroposterior (A-P) RT field (c) Lateral RT field (d) Groin in the last week of RT showing good clinical response (arrow) though skin break down persists probably secondary to RT. The skin healed within 4–6 weeks of completion of RT.

A 44 year old male presented with one year history of slowly enlarging tumor of the right medial thigh and scrotum and extending to the left groin (Figure 1(a)). The lesion was asymptomatic in the beginning and the skin was intact. Gradually the skin over the right inguinal area eroded and the lesion started causing discomfort which became progressively worse. The patient's previous medical history was unremarkable. Laboratory examinations including HIV status were normal. MRI of the right hip showed abnormal fluid-like density ill-defined in nature in the anterior right groin anterior to the pectineus muscle and dissecting into the medial proximal right thigh. There was no clinical or radiological evidence of regional lymph node involvement. Computed tomography scans of the chest and abdomen showed no evidence of metastatic disease. Histological examination of the biopsy specimen revealed numerous anatomizing vascular channels in the dermis. The vascular channels were composed of endothelial cells, some of which had a hobnail appearance. ICC stain for CD31 was positive and HHV8 was negative. Histological diagnosis of hemangioendothelioma with retiform features was established. The patient was initially evaluated for surgical resection but was deemed unresectable because of extent of the disease. A decision was made to treat the patient with chemoradiation and reevaluation after 4140cGy. A 3-dimensional plan was generated to treat the pelvis, after contouring the Gross tumor (based on pretreatment CT and MR images) plus a 2 cm margin (Figures 1(b) and 1(c)). A total dose of 4140cGy in 180cGy per fraction was delivered with an intention of attempt at surgical resection. Weekly Cisplatin (30 mg/m^2^) was given concomitantly with radiation. The patient tolerated the treatment well and had an excellent clinical response (Figure 1(d)). Reevaluation at 4140cGy revealed a minimal radiological response and it was decided to abort treatment at the time because of tumor unresectibility. The patient was followed with a repeat biopsy 8 weeks after RT. Biopsy showed an excellent response but some residual disease. However, a 6-month followup revealed a complete clinical response. The patient has been disease-free at 36 months.

## 3. Discussion

Retiform hemangioendothelioma (RH) is an infrequently encountered vascular neoplasm of intermediate or borderline malignancy that has been classified as a distinct type of low-grade cutaneous angiosarcoma. Up to date, a total of 25 cases were described in the literature [1–11]. First, fifteen cases were described by Calonje et al. in 1994 [1]. It has been proposed by Calonje that in the past examples of the tumor he designated as RH have been diagnosed as angiosarcoma. It is important to distinguish RH from cutaneous angiosarcoma (CA) as the clinical course is quite different. CA can arise in the context of lymphedema (Stewart-Treves syndrome) so can RH (see above) and can be radiation-induced. Cutaneous angiosarcoma has high incidence of recurrence and metastasis and overall carries poor prognosis with high mortality. In comparison, RH has a high local recurrence rate, however, rarely metastasizes and there have been no tumor-related deaths reported up to date [1–11]. Histology is the only way to reliably differentiate RH from CA. Histologically, in CA, the infiltrative pattern is much more disorganized, with sinusoidal or sieve collagen bundles and vascular spaces are more irregular and jagged. Most importantly, even in well-differentiated CA, mitotic figures, cytologic atypia, variation in cellular size, and multilayering can be found [1, 12], while there is almost no cytologic atypia and no mitotic activity in RH.

Morphologically, RH presents as exophytic mass or nodule or a plaque, dermal or subcutaneous, that grows slowly. Most tumors present in young to middle-aged adults (Range 9–78 years) [2]. Most lesions are single though one case of multiple tumors developing in different anatomic sites (trunk and extremities) has been described [3]. One case of regional lymph node metastasis [1] and one soft-tissue metastasis has been reported [4]. There is female predominance 2 : 1 with size range of 1 to 30 cm. Duration of the disease ranged from 2 months to several years. No distant metastases or tumor-related deaths have been reported; however, RH recurs in almost half of the cases. Often, multiple recurrences are observed. Recurrences are observed ranging from months to several years after original presentation. Etiology is unknown; however, an association between RH and human herpes virus type 8 [6], lymphedema [1], previous radiotherapy [1, 6], and noncutaneous malignant neoplasms [1, 6, 7, 11] has been reported but not clearly established. 

Clinically, RH lesions could be located in the dermis (predominantly) with extension into the subcutaneous tissues or striated muscle as was also reported as in our case [1]. A characteristic microscopic feature is the presence of elongated, arborizing, thin-walled blood vessels extending between collagen bundles in a retiform pattern, reminiscent of the architecture of normal rete testis [1]. In some areas, a prominent lymphocytic infiltrate is present. Some areas of the tumor are solid and are composed of epitheloid or spindle cells and dilated vascular channels sometimes with intraluminal papillary projections. Cytologic atypia is minimal and few or no mitotic figures are seen [1, 7]. Immunohistochemically, neoplastic cells are positive for CD 31, CD 34, factor VIII-related antigen, and bound *Ulex earopaeus agglutinin *(UEA) (7). The spindle cells are positive for UEA-1 and CD 31 but not for FVIII-Rag and CD34 [1]. RH may rarely express D2-40, but it does not usually express VEGR-3, markers of endothelium of lymphatic vessels [13]. In summary, based on the staining patterns, it has been suggested that RH is a vascular entity which usually does not have lymphatic differentiation [13].

Other vascular tumors that morphologically can present similarly to RH and need to be differentiated from RH include Dabska's tumor, lymphoma, dermatofibrosarcoma protuberans, hemangioma, bacillary angiomatosis, cutaneous metastases, blue-rubber bleb nevus syndrome, Kaposi's sarcoma, targetoid hemosiderotic hemangioma, hobnail hemangioma, epitheloid hemangioendothelioma, and polymorphous hemangioendothelioma of lymph node [1, 2, 7, 9]. Even though some histological overlap exists, each of the neoplasms has their unique features [1, 2, 8].

 Treatment of choice for RH has been surgical excision with histopathologically tumor-free margins. In the cases reported up to date, patients were treated initially by surgical excision. Approximately 50% of the cases recurred [1–13]. Amputation of hand, disarticulation of toe, partial penectomy, and amputation of finger has been described [1]. Radiotherapy has been reported to be successfully administered as adjuvant treatment for local as well as regional nodal RH recurrences [1, 6]. In addition, adjuvant immunotherapy with recombinant interferon alpha has been tried. As per our knowledge, our report is the first reported successful treatment of RH with low-dose cisplatin and moderate RT without surgical resection. Successful treatment of this uncommon tumor remains under investigation.
